# Perspectives on SARS-CoV-2 Cases in Zoological Institutions

**DOI:** 10.3390/vetsci11020078

**Published:** 2024-02-07

**Authors:** Remco A. Nederlof, Melissa A. de la Garza, Jaco Bakker

**Affiliations:** 1 Independent Researcher, 2861 XZ Bergambacht, The Netherlands; remco.a.nederlof@gmail.com; 2 Michale E. Keeling Center for Comparative Medicine and Research, University of Texas MD Anderson Cancer Center, Bastrop, TX 78602, USA; 3 Biomedical Primate Research Centre, 2288 GJ Rijswijk, The Netherlands

**Keywords:** SARS-CoV-2, zoo, one health, COVID-19, felids, nonhuman primates, artiodactyls, vaccination, diagnostics, long COVID

## Abstract

**Simple Summary:**

Severe acute respiratory syndrome coronavirus 2 (SARS-CoV-2), the virus that caused the COVID-19 pandemic, has infected many different animal species, including those housed in zoos around the world. This review analyzes reports of SARS-CoV-2 infections in zoo animals to understand which species are susceptible and what symptoms may be observed in these species. A variety of diagnostic and sampling methods are discussed, as well as available treatment options. Moreover, this review discusses the factors involved in transmission of the virus and assesses the risk of virus transmission from people to animals and from animals to people. Part of this risk may lie in the occurrence of mutations in different animal species, that could potentially spill back to humans. An emphasis is put on disease monitoring and biosecurity measures in order to minimize the risk of disease to both people and animals.

**Abstract:**

Severe acute respiratory syndrome coronavirus 2 (SARS-CoV-2) infections in a zoological institution were initially reported in March 2020. Since then, at least 94 peer-reviewed cases have been reported in zoos worldwide. Among the affected animals, nonhuman primates, carnivores, and artiodactyls appear to be most susceptible to infection, with the Felidae family accounting for the largest number of reported cases. Clinical symptoms tend to be mild across taxa; although, certain species exhibit increased susceptibility to disease. A variety of diagnostic tools are available, allowing for initial diagnostics and for the monitoring of infectious risk. Whilst supportive therapy proves sufficient in most cases, monoclonal antibody therapy has emerged as a promising additional treatment option. Effective transmission of SARS-CoV-2 in some species raises concerns over potential spillover and the formation of reservoirs. The occurrence of SARS-CoV-2 in a variety of animal species may contribute to the emergence of variants of concern due to altered viral evolutionary constraints. Consequently, this review emphasizes the need for effective biosecurity measures and surveillance strategies to prevent and control SARS-CoV-2 infections in zoological institutions.

## 1. Introduction

Severe acute respiratory syndrome coronavirus 2 (SARS-CoV-2) is the causative agent of coronavirus infectious disease 2019 (COVID-19) and has been circulating in the human population since its emergence in December 2019. SARS-CoV-2 is a single-stranded, positive-sense enveloped RNA virus belonging to the genus Betacoronavirus. The virus lends its tropism to its attachment to the angiotensin-converting enzyme 2 (ACE2) receptor-binding domains of cells, primarily in the lungs, arteries, heart, kidneys, and gastrointestinal tract [1]. The first reported SARS-CoV-2 infection in zoos occurred in tigers and lions at the Bronx Zoo in New York, in March 2020 [2]. Since then, at least 94 peer-reviewed cases have been reported in zoos around the world. Large felids and nonhuman primates were most frequently reported, but natural infection has been observed in other carnivores and artiodactyl species. The severity of disease ranges from asymptomatic infection to death; although, most infections are considered to be mild. Zoos provide a unique challenge with regard to the management of zoonotic infectious disease, as a great diversity of species are kept at population densities that may or may not be representative of the natural situation. Furthermore, human–animal interactions are frequent, and comprise both interactions with zoo staff and the general public. Since late 2020, SARS-CoV-2 evolution has been characterized by the emergence in sets of mutations, in the context of variants of concern (VOC), that impact virus characteristics, including transmissibility and antigenicity [3]. The frequency of spillover events to other species appears to be relatively low, yet different viral evolutionary constraints in animal species have resulted in concerns regarding reservoir formation and the occurrence of new VOCs. As a result, zoological institutions must remain vigilant to protect the health of the animals in their collections, as well as the health of staff and the general public.

## 2. SARS-CoV-2 Host Range and Clinical Disease

### 2.1. Predicted and Demonstrated Susceptibility

At the start of the pandemic, early research on susceptibility focused on in vitro assessment of the ACE2 receptor. These structure-based studies were valuable in predicting how ACE2 orthologues bind to the SARS-CoV-2 viral spike protein receptor-binding domain (RBD) [4,5]. However, the results from these studies have not been consistently supported by experiments [6]. Recent research has focused on investigating the role of transmembrane protease serine 2 (TMPRSS2), which plays a key role in ACE2 cleavage, allowing subsequent SARS-CoV-2 entry into the cell. Analyses integrating both ACE2 and TMPRSS2 may prove to be a better predictor for susceptibility to infection, although more research in this field is required [7].

Experimental infection of animals appears to be a better predictor of susceptibility, although these studies seldom aim to assess a species’ potential role in transmission. More frequently, experimental infection aims to identify animal models for further SARS-CoV-2 research. Experimental viral loads may significantly exceed those that animals may experience during a natural infection, and virions may be directly inoculated intranasally, intratracheally, or intravenously. Successful experimental infection of animals must therefore be interpreted with caution.

Reports of natural SARS-CoV-2 infection, although few in number, remain the best predictor of clinical disease and (reverse) zoonotic risk. These reports must, however, be considered in light of the nature of the human–animal contact and the prevalence of SARS-CoV-2 in the area at the time of infection. Table 1 provides an overview from the literature of experimental and natural SARS-CoV-2 infections in species commonly housed in zoological institutions.

### 2.2. Carnivores

#### 2.2.1. Lions

Lions (*Panthera leo*) represent the biggest group (*n* = 37) of reported cases. Cases have been reported in both the Asiatic [36,37,38,39] and African subspecies [2,28,31,32,33,35]. The susceptibility of this species is underpinned by the infection of vaccinated (1.0 mL SQ or IM, Zoetis International, Parsippany, NJ, USA) animals and by reinfection of recovered animals [28,31]. Almost all (96.4%) cases were symptomatic, with symptoms lasting 14.8 days on average (Table 2). Although in one case, coughing was observed to persist for 49 days [31]. Coughing is reported in 81.5% of cases and is the most frequently observed clinical sign. Less frequently, lethargy was observed in 55.6% of cases (Table 2). Epistaxis is infrequently reported but may be associated with SARS-CoV-2 infection, as it has also been observed in infected tigers (*Panthera tigris*) and a leopard cat (*Prionailurus bengalensis*) [34,37,39]. Although disease is generally considered to be mild in lions, two SARS-CoV-2 related deaths have been reported [38].

#### 2.2.2. Tigers

SARS-CoV-2 infections are relatively frequently observed in tigers (*n* = 24) [2,28,31,34,37,41,42,43,44]. Similar to lions, infections have been reported in vaccinated (1.0 mL SQ or IM, Zoetis International) animals [28]. Interestingly, confirmed intraspecies transmission further corroborates this species’ susceptibility to SARS-CoV-2 infection [43]. Almost all (95.0%) cases were symptomatic, with clinical signs lasting 10.2 days on average. Similar to lions, coughing was the most frequently observed clinical sign (94.7%), followed by lethargy (63.2%). Hyporexia or anorexia was observed in 52.3% of cases, similar to what has been reported in lions. In contrast, whereas wheezing was infrequently observed in lions (14.8%), it was commonly observed (63.2%) in tigers. Discharge was infrequently observed, with nasal discharge (36.8%) being more commonly reported than ocular discharge (15.8%). Furthermore, sneezing, vomiting, diarrhea, and epistaxis have been infrequently reported (Table 2). Although tigers generally appear to be afflicted more severely by SARS-CoV-2 infection than lions, no deaths have been reported in this species [37]. 

#### 2.2.3. Other Felids

Snow leopards (*Panthera uncia*) are considered to be highly susceptible to severe SARS-CoV-2 infection (*n* = 6). Although no SARS-CoV-2 related deaths have been described in the peer-reviewed literature, non-peer-reviewed reports suggest a high sensitivity of this species. Of the six reported peer-reviewed cases, five animals displayed clinical symptoms [28,45]. Again, coughing was observed most frequently (60.0%), followed by hyporexia (40.0%), and wheezing (40.0%). Lethargy and diarrhea were observed infrequently, in only one case each (Table 2). Low genetic diversity in snow leopards may contribute to their apparent susceptibility to SARS-CoV-2, although additional research is needed on this relationship [67].

Although infections in both the European lynx (*Lynx lynx*) and Canadian lynx (*Lynx canadensis*) have been reported, only the Canadian lynx SARS-CoV-2 infections have been described in the peer-reviewed literature (*n* = 4) [20,31]. All of the infected animals were symptomatic, with clinical signs lasting between 17 and 35 days. Lethargy and hyporexia were seen in all lynxes, with three of the lynxes also displaying a cough. Notably, diarrhea was noted in one lynx, which is infrequently reported in other species [31].

Only a single report of infection in a leopard cat (*Prionailurus bengalensis*) exists [37]. Symptoms lasted for two days and consisted of sneezing, nasal discharge, diarrhea, and epistaxis.

Only one case in a fishing cat (*Prionailurus viverrinus*) has been described. Despite being vaccinated (1.0 mL SQ or IM, Zoetis International), the animal displayed lethargy, hyporexia and diarrhea [28].

Early on in the pandemic, SARS-CoV-2 cases in cougars (*Puma concolor*) were reported (*n* = 2). Symptoms lasted 22.5 days on average, and consisted of nasal discharge, hyporexia, and diarrhea in both animals. Additionally, coughing was observed in one animal [33].

Leopard (*Panthera pardus*) SARS-CoV-2 cases have not yet been reported in zoological institutions; however, a 7.9% seroprevalence was found in a retrospective survey on wild leopards in India [40]. Whilst this suggests susceptibility to infection, no statements about clinical disease can be made at this time.

#### 2.2.4. Mustelids

The initial outbreak involving American mink (*Neovison vison*) took place within mink farms located in the Netherlands in 2020 [27]. Subsequently, additional outbreaks were documented in various countries, with Denmark experiencing the most severe impact [25]. Mink appear to be effective amplifiers and transmitters of SARS-CoV-2, as mink-to-mink transmission following reverse zoonotic infection is the most plausible explanation for these outbreaks [27]. Neither clinical signs nor increased mortality were observed by approximately one-third of mink farmers in Denmark. Reduced feed intake (>20% of mink), respiratory symptoms, and nasal discharge (<1% of mink) were reported most frequently. Clinical signs lasted anywhere between 0 and 33 days, with a median of 11 days. Increased mortality was observed on 63% of farms, with a median daily mortality rate of 0.14% as opposed to a normal weekly mortality rate of 0.008% [25]. Mink are infrequently kept in zoological institutions, and usually at population densities significantly smaller than in farmed settings, decreasing the risk of spillover.

Whereas infection in captive Asian small-clawed otters has only been reported in non-peer reviewed reports, wild Eurasian river otters (*Lutra lutra*) have been observed to be naturally susceptible to SARS-CoV-2 infection [20,21]. No information about clinical disease or intraspecies transmission is available in this species, as only one case was reported following necropsy of a road-killed specimen. Nevertheless, this highlights the importance of viral surveillance in wild mustelids [21]. 

#### 2.2.5. Procyonids and Viverrids

During an outbreak event in a zoo, vaccinated (1.0 mL SQ or IM, Zoetis International) white-nosed coatis (*Nasua narica*) tested positive on RT-PCR (*n* = 2), but no symptoms were observed [28].

Two vaccinated (1.0 mL SQ or IM, Zoetis International) binturongs (*Arctictis binturong*) tested positive on RT-PCR, although symptoms were only observed in one individual. The symptomatic binturong displayed signs of lethargy and hyporexia. Although symptoms were reportedly mild, significant pathological change in the lungs were detected by a CT scan [28]. 

### 2.3. Nonhuman Primates

#### 2.3.1. Old World Primates

Early in silico analysis, Old World primates were flagged as highly susceptible to SARS-CoV-2 infection, based on ACE2-receptor configuration [5]. However, the overall number of natural infections of nonhuman primates appears to be relatively low.

A total of twelve cases of natural SARS-CoV-2 infection in Western lowland gorillas (*Gorilla gorilla*) have been described [36,37]. This species’ susceptibility to natural infection is further corroborated by the observation of intraspecies transmission [37]. Symptoms were observed in 81.7% of gorillas, with clinical signs lasting 10.4 days on average. Similar to non-primate species, coughing was frequently observed (90.9%). Notably, nasal discharge was observed in all affected individuals. Furthermore, 72.7% of animals displayed signs of hyporexia and 41.7% were observed to be lethargic (Table 2).

#### 2.3.2. New World Primates

Whilst the susceptibility of New World monkeys has been a topic of uncertainty, evidence has surfaced through both the peer reviewed and non-peer reviewed literature describing natural SARS-CoV-2 infection in a common squirrel monkey (*Saimiri* sp.), common woolly monkey (*Lagothrix lagothricha*), black-headed spider monkey (*Ateles fusciceps*) and a black-tailed marmoset (*Mico melanurus*) [20,52]. Still, more research is required to characterize clinical disease and the potential for intraspecies transmission in these species.

### 2.4. Artiodactyla

#### 2.4.1. Hippopotamuses

The infection of captive hippopotamuses (*Hippopotamus amphibius*) (*n* = 3) emphasizes that structural analysis of the ACE2-receptor alone is insufficient to predict a species’ susceptibility to infection, as they were predicted to be at medium risk [5,13,14,65]. Overall, three hippopotamus cases have been described [13,14]. All hippopotamuses showed clinical symptoms, with nasal discharge being most frequently reported [14]. Only one hippopotamus died due to SARS-CoV-2 infection 17 days after the onset of clinical symptoms. The primary finding of the necropsy was a severe pneumonia [13]. 

#### 2.4.2. Cervids

In spite of the shortcomings of in silico analysis, white-tailed deer (*Odocoileus virginianus*) were predicted to be highly susceptible to SARS-CoV-2 infection [5]. Multiple distinct SARS-CoV-2 lineages recovered in white-tailed deer provide evidence for multiple spillover events [68]. Additionally, sustained horizontal transmission has been reported in wild white-tailed deer [12,68,69]. There is little concern about the impact of SARS-CoV-2 on white-tailed deer, as only a transient and modest increase in body temperature was reported in experimentally infected fawns [70]. Nevertheless, white-tailed deer have demonstrated effective viral transmission and potential for reservoir formation, and precautionary measures around this species in zoological settings should be considered accordingly.

Other than white-tailed deer, seropositivity has been observed in wild fallow deer (*Dama dama*) and red deer (*Cervus elaphus*) in Europe. Spillover likely occurred due to human-to-deer transmission, but it is unknown whether intraspecies transmission plays a role in these species [9].

## 3. Viral Dynamics and Diagnostics

### 3.1. Viral Dynamics

Viral dynamics in animals appear to be similar to those seen in humans, with viral shedding peaking around the time of onset of clinical symptoms and rapidly decreasing afterwards [28,71]. In most lion cases, the shedding of viral RNA was undetectable within 20 days but may be as long as 53 days [28]. The human literature reports a median clearance for viral RNA at 16 days post onset of clinical signs [71]. Symptoms in lions have been reported to appear as late as 11 days after the onset of detectable viral shedding, but generally appear within a shorter timeframe [31]. Symptoms in lions persist for an average of 14.8 days (Table 2), which is longer than the average of ten days reported in humans [71]. It has been reported that symptomatic animals remain positive on real-time PCR longer than asymptomatic animals, which may help guide monitoring during outbreaks [28]. Similarly in humans, the RNA viral load is comparable between mild and severe cases, but patients with severe disease show elevated RNA loads in the second week of infection and RNA can be detected for prolonged periods [72]. Across reported animal cases, intermittent shedding has been observed in 73.3% of cases (Table 3).

### 3.2. Diagnostic Methods

#### 3.2.1. RT-PCR

Virus-specific (semi-)quantitative RT-PCR on respiratory tract samples is regarded as the gold standard for SARS-CoV-2 diagnosis [71]. A major advantage of RT-PCR is that there are no cross reactions with non-SARS-CoV-2 coronaviruses, resulting in high specificity. Quantitative RT-PCR yields a CT (cycle threshold) value, which is inversely correlated with viral RNA load in the sample. CT values greater than 34 have been determined to be non-contagious in humans [73]. A positive RT-PCR does not necessarily imply infectiousness, as it cannot differentiate between replication-competent virus and non-infectious residual viral RNA. As a result, it has been suggested that the correlation between infectious virus and RNA viral load, using CT values as a proxy, is low, making CT values a poor predictor of infectious virus presence in the first five days post-onset of clinical symptoms [74]. 

The emergence of variant SARS-CoV-2 strains must also be considered, as novel mutations may render specific PCR primers ineffective. Multiplex PCR tools can overcome this potential issue and must be used in order to reliably diagnose animals with variant SARS-CoV-2 strains. The Nucleocapsid and Envelope sequences are deemed most stable, and therefore, serve as suitable targets for RT-PCR [75].

#### 3.2.2. Virus Isolation

Although practical reasons often preclude virus isolation as a primary means to reach a diagnosis, it is considered the gold standard to demonstrate the presence of infectious viral particles in a sample [71]. Virus isolation has been effectively used in multiple reports to verify a SARS-CoV-2 diagnosis [2,13,35,39,44]. The probability of a successful culture in humans has been demonstrated to rapidly decline after eight days post-onset of clinical signs, and virus has not been isolated from seroconverted patients with detectable antibody titers [76,77]. 

#### 3.2.3. Antigen Tests

A rapid antigen detection test (RADT) is a type of lateral flow test that detects the presence of the viral nucleocapsid (NP) protein. RADTs have been reportedly used in five zoological cases, all yielding positive test results [32,35]. Antigen tests have demonstrated to have higher specificity, as high as 98% in humans, yet lower sensitivity than PCR tests [78]. Especially when CT values are below 25–30, which is likely during the period in which infectious virus is present, RADT detections show good concordance with RT-PCR positivity [79]. In contrast, RADTs are less reliable during periods of infection where RT-PCR CT values are high [80]. Furthermore, it has been suggested that antigen test positivity might be a better marker of infectiousness than a positive RT-PCR result [81]. As a result, RADTs may prove useful following a positive RT-PCR to monitor risk of transmission [35]. 

#### 3.2.4. Serology

A double antigen sandwich ELISA detects both IgM and IgG antibodies. Various viral antigens may be used as targets, and multiplex assays can be created to test for different antigens simultaneously [82]. The ELISA that targets antibodies against the SARS-CoV-2 N-protein has been validated according to different animal species, notably mink, tigers and leopard cats among others, for cross-reactions with other coronaviruses [82]. Nevertheless, one must be wary when interpreting unverified positive serological results, as many animal species already harbor various species-specific coronaviruses [82]. Serology appears to be a reliable means of demonstrating past infection with SARS-CoV-2. Across the 14 serological tests performed in reported zoo cases, only one negative result was obtained in a gorilla. The authors hypothesized that this was most likely due to blood sampling occurring only five days post-onset of clinical signs. No blood has been collected at a later date for further analysis [36]. Yet, in tigers, a positive serological test was observed as early as six days post onset of clinical symptoms [2]. Whilst an approximated 30–40% seroprevalence has been reported in wild white-tailed deer in the US with unknown exposure [83,84], a seroprevalence of 94.4% was observed following an infection in a captive setting [11]. Similarly, following the outbreak in American mink in Denmark, seropositivity was observed to be 100% in tested farms [25]. Although it is unknown how long antibodies persist in most species, in white-tailed deer, antibodies have been shown to persist for at least 13 months, with few individuals (~10%) having antibodies wane below detectable levels within this timeframe [11]. 

### 3.3. Sampling Types

#### 3.3.1. Oral and Nasal Samples

In humans, nasopharyngeal samples are considered to be the gold standard, with pooled nasal and throat swabs being the best performing alternative sampling method [71]. There appears to be a variable correlation between oral and nasal viral loads in big cats, with nasal viral loads appearing to be higher overall [42]. Across reported species, 96.8% of nasal RT-PCR tests had a positive result (Table 4), with only a single tiger testing negative [42]. Although the sampling size is small, only three out of five cases in which oral RT-PCR test was performed reported a positive test result (Table 4). A total of two tigers tested negative on oral samples whilst simultaneously testing positive on either fecal or nasal samples [42]. In spite of this apparent reduced reliability of oral sampling, repeated oral swabbing tends to be better tolerated by trained felids than nasal swabbing, facilitating sample collection [42]. Throat swabs can be considered as postmortem samples or may be obtained from animals under anesthesia. This method has been infrequently reported, although during the peak of infection in mink in Denmark, 96% of throat swabs tested positive by RT-PCR [25].

#### 3.3.2. Fecal and Rectal Samples

The major practical advantage of fecal sampling is that it is non-invasive. Fecal shedding of viral RNA is reported to last longer than oronasal shedding [28,45]. It has been demonstrated that viral RNA can still be detected in the feces one month after the onset of clinical signs in lions [33]. Others report as little as two weeks after the onset of clinical signs before viral RNA shedding becomes undetectable [35]. Intermittent shedding is frequently observed in fecal samples across species [14,28,34,35,42,45]. Intermittent periods in fecal shedding of viral RNA of up to 25 days have been described in tigers [34]. Fecal detection of viral RNA may be less sensitive than respiratory sampling and may not parallel respiratory disease. During the early stages of infection, fecal shedding may be negative, whereas during later stages of infection, viral RNA may be demonstrated in absence of infectious virus [35]. Across reported SARS-CoV-2 cases in different zoological institutions, 82.1% of fecal PCR tests were reported to be positive (Table 4). In contrast, in spite of being reported less frequently, all reported PCR tests performed in lions and tigers on rectal swab samples had a positive result [38,39,42]. 

#### 3.3.3. (Waste) Water Sampling

Monitoring of pool water provides a non-invasive way of monitoring viral dynamics. A total of 53% of pool water samples were observed to be positive on PCR in a case of SARS-CoV-2 in a captive hippopotamus. This was in contrast to only 27% of the combined fecal and nasal samples testing positive during the same period. The presence of viral RNA in the pool water is primarily attributed to the constant exchange of nasal fluid with the water in semi-aquatic species, although it must be mentioned that the demonstration of viral RNA does not necessarily imply the presence of infectious virus [14].

Water analysis may also prove useful for mass surveillance strategies. Wastewater can be routinely tested for viral RNA to monitor the presence of SARS-CoV-2 in staff and animals in a facility [85]. 

#### 3.3.4. Tissue Samples

Tissue RT-PCR was performed on a deceased hippopotamus, and the lowest CT value was reported in the lungs (27.09), followed by the liver (32.29), spleen (33.96), and intestine (37.84), whilst postmortem RT-PCR on blood was negative [13]. Similarly, RT-PCR prevalence of SARS-CoV-2 in retropharyngeal lymph nodes was determined to be as high as 81.3% in wild white-tailed deer, shortly following a peak of infections in humans in the area [68]. Although tissue samples are rarely accessible to the clinician, they should not be overlooked as a means to confirm a diagnosis if available.

## 4. Treatment Options

Natural SARS-CoV-2 infection in animals is commonly associated with mild clinical symptoms. As such, supportive care consisting of non-steroidal anti-inflammatory therapy or low-dosage glucocorticoid therapy, vitamin supplementation, mucolytics, and fluid therapy are likely to alleviate symptoms for the duration of the infection. In the case of severe respiratory distress, albuterol and oxygen therapy may be considered [86].

Antiviral therapy in animals has only been applied in experimental studies. Remdesivir has been demonstrated to reduce clinical signs of disease in experimentally infected rhesus macaques (*Macaca mulatta*); however, no reduction in viral shedding was observed [87]. Similarly, the usage of lopinavir-ritonavir, hydroxychloroquine sulfate, and emtricitabine-tenofovir marginally reduced clinical disease and only the emtricitabine-tenofovir treated group displayed reduced viral shedding [88]. Therefore, the administration of antiviral drugs is not currently recommended in natural SARS-CoV-2 infections across species. 

Monoclonal antibody therapy has been applied in human COVID-19 cases and poses a promising therapy in animals, as human-neutralizing antibodies have shown to protect Syrian hamsters (*Mesocricetus auratus*) in an experimental infection [89]. Moreover, the non-peer reviewed literature reports the successful use of this therapy in an older gorilla [90]. Future application of this therapy will likely depend on the availability of monoclonal antibodies, as well as both legal and financial restraints.

Antimicrobial therapy is not recommended for the treatment of SARS-CoV-2 in animals and should only be considered for the treatment of secondary infection. Whereas some antimicrobial drugs have shown promise in vitro, these results are not supported by in vivo studies [91].

## 5. Viral Transmission and Associated Risks

### 5.1. SARS-CoV-2 Transmission

#### 5.1.1. Surface Contamination, Stability, and Disinfection

Viral stability within the environment is governed by a multitude of factors. Viable virus has been isolated for up to 3 hours from aerosols and up to 72 h from surfaces [92]. SARS-CoV-2 is sensitive to heat, with reported inactivation within five minutes at 70 °C but exhibits markedly increased stability at lower temperatures [93]. On cold-chain food packaging kept below −18 °C, SARS-CoV-2 has been demonstrated to persist for at least 60 days [94]. Furthermore, low environmental moisture tends to accelerate SARS-CoV-2 inactivation [95]. Surface material and texture may play a significant role in the survival of virus particles [96]. A protein-rich medium, such as airway secretions, may protect the virus and extend the longevity of infectious virus in the environment, increasing the likelihood of fomite transmission [97]. In contrast, SARS-CoV-2 is readily inactivated by disinfectants [93,98]. Additionally, exposure to UV-C light will inactivate SARS-CoV-2 virus over time, although the rate of inactivation depends on the wavelength used and exposure time [99].

Transmission can be limited by wearing the appropriate protective personal equipment (PPE), depending on the activity to be performed. Activities that entail a higher level of contact with animals require increased PPE, such as surgical masks, gloves, face masks, and goggles. Always wear new, or cleaned and disinfected PPE such as masks, aprons, gloves, and boots when moving between enclosures. Use footbaths with clean disinfectant, such as Virkon-S, to disinfect boots when entering and leaving enclosures. Routinely clean and disinfect common areas for caretakers, e.g., resting areas, kitchen, coffee room, changing rooms, and bathrooms. Avoid rotating workers among different zoo sections, as doing so will increase the potential of virus spread. Ensure physical distance between people is observed at all times, at least 1.5 m, at times of high SARS-CoV-2 occurrence. Stagger mealtimes and breaks to avoid large gatherings in the break rooms. Prepare for a possible shortage of the workforce and prepare a contingency plan to ensure continuity of work. When using tools, make sure to always disinfect them before use and after use. Basic personal hygiene measures should be practiced, in particular regular handwashing before and after handling animals. Raise awareness among caretakers about how SARS-CoV-2 spreads and how to avoid becoming infected, and routinely remind them about biosafety and biosecurity measures to protect against SARS-CoV-2. In case of animal transfer, use an all-in all-out strategy. Ensure that appropriate cleaning and disinfection is performed before restocking, using the recommended disinfectants and following the instructions on the product label [100].

#### 5.1.2. Methods of Transmission

Droplet transmission rather than aerosol transmission has been established as the most important mode of transmission [101]. This is corroborated in animals by the positivity of RT-PCR samples collected within one meter of animal cages on mink farms in Denmark [102]. Additionally, poor ventilation has been implicated in multiple transmission events [103]. In mink, airborne transmission was suspected based on the detection of viral RNA in airborne-inhalable dust, likely contaminated with feces [27]. Although the finding of SARS-CoV-2 RNA in feces by PCR does not necessarily equate to the presence of an intact viable virus, virus isolation has demonstrated the presence of viable virus in the feces of nondomestic felids [34]. Furthermore, especially in zoological settings, fecal matter may become aerosolized during routine cleaning procedures [42]. In human medicine, the fecal–oral transmission of SARS-CoV-2 still constitutes a major knowledge gap. Cell, organoid, and animal infection by SARS-CoV-2 from feces have been observed but no strong evidence of human SARS-CoV-2 fecal–oral transmission was found. Therefore, the risk of disease transmission through the fecal–oral route cannot be excluded [104,105].

Other than aerogenic spread, fomites may inadvertently introduce SARS-CoV-2. As such, contaminated bedding material is implicated in the transmission of SARS-CoV-2 among farmed mink [27]. 

In the case of mink, the exchange of personnel between farms was identified as a risk factor, either because the personnel were infected or because they may have served as fomites [106]. In fact, in Denmark, direct human contact was identified as the most probable method of virus transmission between farms [25]. Likely infection by asymptomatic personnel has also been implicated in case reports in zoological settings [36].

Under normal circumstances, SARS-CoV-2 is unlikely to be transmitted through sewage or natural water systems due to short viability and dilution of the virus, although high human or animal population densities, poor sanitation, faulty plumbing, and direct contact with fresh sewage within a theoretical one-and-a-half-day viability timeframe could pose a threat [85]. Water may play a more important role in (semi-)aquatic species, as viral RNA has been recovered from the pool water of hippopotamuses [14]. Additionally, waterborne transmission may have played a role in the infection of in a wild European river otter [21]. It remains uncertain whether life support sterilization units effectively remove infectious material from pool water, based on studies on human wastewater treatment [14,107]. 

### 5.2. Spillover, Mutations, and Variants

#### 5.2.1. Spillover to Humans

Zoonotic transmission following reverse zoonotic infection has been reported in lions, mink, and white-tailed deer, with sustained horizontal transmission having been observed in mink and white-tailed deer [11,26,32,106]. Wild white-tailed deer constitute a potential natural SARS-CoV-2 reservoir [11,32,106]. In captivity, however, restraints in population size and frequent monitoring reduce the overall chance of animal reservoirs occurring and reduce the subsequent risk in the emergence of novel variants.

#### 5.2.2. Variants of Concern

VOCs are SARS-CoV-2 strains that show an increased transmissibility compared to the original virus and have the potential to increase disease severity [108]. The pattern of viral load dynamics is conserved between variants, but differences in infectious virus load, RNA viral load, and incubation period have been observed [109,110]. New SARS-CoV-2 variants are unlikely to have a large effect on molecular diagnostics, as dual-target assays detect at least two viral genes simultaneously [71]. Although diagnostics are minimally affected, VOCs exhibit decreased susceptibility to vaccine-induced and infection-induced immune responses and may thus possess the ability to reinfect previously infected and recovered individuals, as has been demonstrated in lions, where infections with both the alpha and delta variants have been demonstrated in the same individuals [31,108].

Concerns exist over the occurrence of new VOCs spilling over from animal reservoirs into the human population. Mutations that occur infrequently in humans, especially those in the receptor binding motif, may be amplified in new host species with different constraints on viral evolution [12]. Furthermore, the risk of a variant emergence may be increased by heightened evolutionary rates, such as those reported in mink and white-tailed deer [69,106]. Following infection in Asiatic lions, mutations not observed in the human population have been reported, although their relevance with regard to transmissibility and pathogenicity remain unknown [38]. A large outbreak in farmed mink in Denmark led to the infection of approximately 4000 human cases with the mink variant, which was reportedly less susceptible to neutralization by antibodies acquired through prior infection [26].

Conversely, although many species have demonstrated a lack of SARS-CoV-2 susceptibility, spillover into animals may be enhanced through expansion of SARS-CoV-2 host cell tropism. For example, house mice (*Mus musculus*) previously demonstrated to be resistant to wildtype SARS-CoV-2 infection, have been shown to be susceptible to infection by the alpha variant [58]. Comparative analysis of nucleotide sequences in identified SARS-CoV-2 strains can yield valuable insights. Examining the molecular characteristics of SARS-CoV-2 isolates originating from both humans and animals can facilitate the construction of informative phylogenetic trees, which may allow for better prediction of susceptibility in animal species [111]. Moreover, examination of presumed amino acid alterations in the viral proteome across diverse species may reveal significant insights [3,112].

Overall, the risk of VOC development in zoological settings is considered to be low [36]. Nevertheless, the different evolutionary constraints imposed on the virus by different species, as well as the high human–animal contact frequency, warrant some degree of caution with regard to spillover of infrequently occurring mutations.

## 6. Moving Forward

### 6.1. Early Detection Strategies

Active surveillance of individual animal samples may be costly and unrewarding. Seropositivity was observed in 6.5% of tigers in a zoological institution in Thailand; although real-time PCR was negative, implicating past asymptomatic infection [41]. Similarly, fecal surveillance in two Flemish zoos between 2020 and 2021 using real-time PCR in fecal samples yielded no positive results other than two previously diagnosed hippopotamuses [65]. Surveillance should be considered during periods with high human incidence of disease in the area and should focus on the susceptible animal species. Collective fecal samples and wastewater analysis may prove useful in monitoring the occurrence of SARS-CoV-2 in zoological institutions. Monitoring can also be considered in light of its epidemiological purpose, as the creation of a serobank may allow for backtracing of previously undetected infection [69].

### 6.2. Biosecurity and Visitor Management

To limit the risk of SARS-CoV-2 transmission in zoos, an important first step for clinicians is to recognize SARS-CoV-2 as a differential diagnosis in susceptible species displaying relevant clinical symptoms. 

Furthermore, PPE should be provided to all staff working directly with susceptible animal species. A pattern can be recognized across reported zoo cases, where lack of PPE availability was mentioned in 32.7% of cases (Table 3), and improper PPE usage was suggested by others [36]; this in spite of surgical masks being reportedly effective when tested on animals [113]. Along with the usage of PPE, contact time with animals should be reduced where possible.

Zoo staff may be encouraged to receive SARS-CoV-2 vaccinations in light of reducing the risk of both zoonotic and reverse zoonotic infections occurring. In cases where vaccination was mentioned, less than half (43.8%) of keepers were reported to have been vaccinated at the time of reverse zoonotic infection (Table 3). 

The chances of spillover may be further reduced by physical barriers, such as glass walls, and appropriate distancing between animals and humans. To further reduce potential transmission to animals, enrichment constructed out of anthropogenic items should be disinfected to allow a potential surface viral load to decrease.

Indoor ventilation systems should not expose animals to unfiltered air that has been in staff or visitor areas prior. Air filters should be HEPA type or greater, and UV–treatment of passing air may also aid in reducing the viral load. Increasing air change rate was observed to the most effective protective measure regarding ventilation systems. The air renewal volume should preferably exceed ten changes per hour with the fraction of fresh air at all times exceeding 0.3 [114].

Lastly, following confirmed infection of staff members, quarantine procedures should be in place, in spite of mandatory quarantine periods having been lifted in most of the world. Similarly, virus containment protocols should be in place in case an animal displays clinical symptoms associated with SARS-CoV-2.

### 6.3. Considerations for Vaccination Programs

Worldwide efforts to vaccinate the human population have helped manage the SARS-CoV-2 pandemic. The first SARS-CoV-2 vaccine developed for animals, Karnivak-Kov, has been designed for carnivores. Reportedly, immunity lasts at least six months after vaccination [115]. This vaccine, however, has not found widespread application. 

One paper describes the vaccination of zoo animals with an experimental S-peptide based recombinant SARS-CoV-2 vaccine (1.0 mL SQ or IM, Zoetis International), aiming to reduce the severity and mortality of potential SARS-CoV-2 infections in animals. The vaccination schedule was designed to occur in phases, prioritizing felids and primates based on proposed susceptibility [5,28]. Animals were vaccinated with a two-dose series, separated by at least three, but no more than five weeks. No information is available on the efficacy of the vaccine in nondomestic animal species, although protective immunity is likely not reached in all animals, as natural infection following vaccination has occurred [28]. Following reverse zoonotic infection in a vaccinated lion, subsequent zoonotic infections to vaccinated zookeepers have occurred, although the immune status of this particular animal may have been compromised [32].

Neutralizing antibodies have been associated with a reduction of clinical symptoms and viral shedding in felids, suggesting that vaccines might be beneficial in attenuating the severity of a subsequent infection [31]. Furthermore, it is thought that hybrid immunity, that is, vaccination combined with one or multiple natural infections, may provide better control of virus replication in the mucosa than protection acquired solely through vaccination [116].

For most zoological species, the combination of mild clinical symptoms and a relatively low occurrence of reverse zoonotic SARS-CoV-2 infection reduces the urgency of vaccination. However, vaccination may be considered in susceptible species, such as snow leopards, or species that are at heightened risk of causing zoonotic infection, such as lions and white-tailed deer. From a conservation point of view, vaccination should also be considered in endangered species that are susceptible, or whose susceptibility to SARS-CoV-2 is unknown, in order to minimize the risk of disease occurring in genetically valuable animals. One must also consider whether vaccination is desirable from a practical point of view. The stress imposed on untrained animals and hazards associated with restraint must be weighed against the expected efficacy of the vaccine, the current risk of viral exposure, and patient comorbidity factors.

Lastly, it should be noted that various vaccines are available against coronaviruses other than SARS-CoV-2, e.g., porcine, bovine, feline, canine, and avian coronaviruses. There is no current evidence for cross-immunity provided by these vaccines against SARS-CoV-2. Furthermore, some of the aforementioned vaccines are live attenuated vaccines and may present a disease risk with off-label use in zoological species [117]. 

### 6.4. Long COVID

Currently, post-acute sequelae of COVID-19 (PASC), or long COVID, is a debilitating illness that occurs in approximately 10% of human SARS-CoV-2 infections. Multiple organ systems are affected leading to a wide range of symptoms including fatigue, shortness of breath, changes in smell or taste, insomnia, mental health problems, headache, and cognitive dysfunction [118]. Whilst no reports on long COVID in zoological institutions have been published, it is possible that long COVID in animals is underdiagnosed because symptoms may be subtle and difficult to observe in zoo and wild animal species. The World Health Organization (WHO) defines long COVID as the continuation or development of new symptoms three months after initial infection, with these symptoms lasting for at least two months [119]. Consequently, long COVID symptoms in animals may be misattributed, as their occurrence extends beyond the expected timeframe typically associated with COVID-19. Retrospective research may further our understanding of the potential occurrence of long COVID in animals.

## 7. Conclusions

This review summarizes the current state of knowledge on SARS-CoV-2 infections across a wide range of animal species in zoological settings. The known host range, clinical presentations, diagnostic approaches, and treatment options are discussed. Additionally, factors influencing viral transmission and the risks of spillover events are considered. SARS-CoV-2 is zoonotic in origin, and the detection of SARS-CoV-2 in pet, zoo, and farm animals has highlighted its potential for reverse zoonosis. Human-to-animal, animal-to-animal, and animal-to-human transmission events were suggested in different outbreaks involved in animal infection with SARS-CoV-2, warranting continued testing and surveillance. Whilst variant emergence remains a concern, the likelihood of this occurring in a zoological setting is small. In general, surveillance and strengthened biosecurity protocols can help mitigate risks to animal and human health. Continued research is needed to better understand the susceptibility of a variety of animal species, and to understand long-term impacts of SARS-CoV-2 on animals.

## Figures and Tables

**Table 1:** An overview of reported experimental and natural SARS-CoV-2 infections in animals.

Order	Suborder	Family	Species	Experimental Infection	Source	Natural Infection	Source
Artiodactyla	Ruminantia	Bovidae	Cattle (*Bos taurus*)	X	[8]		
		Cervidae	Red deer (*Cervus elaphus*)			X	[9]
			Fallow deer (*Dama dama*)			X	[9]
			Mule deer (*Odocoileus hemionus*)	X	[10]		
			White-tailed deer (*Odocoileus virginianus*)			X *	[11,12]
	Whippomorpha	Hippopotamidae	Hippopotamus (*Hippopotamus amphibius*)			X *	[13,14]
	Suina	Suidae	Domestic swine (*Sus domesticus*)	X	[15]		
Carnivora	Caniformia	Canidae	Dog (*Canis lupus familiaris*)	X	[16]	X *	[17]
			Raccoon dog (*Nyctereutes procyoniodes*)	X	[18]		
		Mephitidae	Striped skunk (*Mephitis mephitis*)	X	[19]		
		Mustelidae	Small clawed otter (*Aonyx cinereus*)			X *	[20]
			Eurasian River Otter (*Lutra lutra*)			X	[21]
			European pine marten (*Martes martes*)			X	[22]
			European badger (*Meles meles*)			X	[22]
			Ferret (*Mustela putorius*)	X	[23]	X *	[24]
			American mink (*Neovison vison*)			X *	[25,26,27]
		Procyonidae	White-nosed coati (*Nasua narica*)			X *	[28]
			Raccoon (*Procyon lotor*)	X	[19]		
	Feliformia	Felidae	Domestic cat (*Felis catus*)	X	[16,29]	X *	[30]
			Canada lynx (*Lynx canadensis*)			X *	[31]
			Eurasian lynx (*Lynx lynx*)			X ^=^	[20]
			Lion (unspecified) (*Panthera leo*)			X *	[28,32,33]
			Lion (*Panthera leo krugeri*)			X *	[2,31,34]
			Lion (*Panthera leo melanochaita*)			X *	[35]
			Lion (*Panthera leo persica*)			X *	[36,37,38,39]
			Leopard (*Panthera pardus*)			X	[40]
			Tiger (*Panthera tigris*)			X *	[41]
			Siberian tiger (*Panthera tigris altaica*)			X *	[2,28,31,34]
			Malayan tiger (*Panthera tigris jacksoni*)			X *	[2,34,37,42,43,44]
			Sumatran tiger (*Panthera tigris sumatrae*)			X *	[37]
			Snow leopard (*Panthera uncia*)			X *	[28,45]
			Amur Leopard cat (*Prionailurus bengalensis euptilurus*)			X *	[37]
			Fishing cat (*Prionailurus viverrinus*)			X *	[28]
			Cougar (*Puma concolor*)			X *	[33]
		Hyaenidae	Spotted hyena (*Crocuta crocuta*)			X *	[20]
		Viverridae	Binturong (*Arctictis binturong*)			X *	[28]
Chiroptera	Yinpterochiroptera	Pteropodidae	Egyptian fruit bat (*Rousettus aegyptiacus*)	X	[46]		
Cingulata		Chlamyphoridae	Big hairy armadillo (*Chaetophractus villosus*)			X ^=^	[47]
Lagomorpha		Leporidae	European rabbit (*Oryctolagus cuniculus*)	X	[48]	X *	[49]
Pilosa	Vermilingua	Myrmecophagidae	Giant anteater (*Myrmecophaga tridactyla*)			X	[50]
Primates	Haplorhini	Atelidae	Common woolly monkey (*Lagothrix lagothricha*)			X ^=^	[20]
			Black-headed spider monkey (*Ateles fusciceps*)			X ^=^	[20]
		Callitrichidae	Common marmoset (*Callithrix jacchus*)	X	[51]		
			Black-tailed marmoset (*Mico melanurus*)			X	[52]
		Cebidae	Common squirrel monkey (*Saimiri* sp.)	X	[53]	X *	[20]
		Cercopithecidae	African green monkey (*Chlorocebus aethiops*)	X	[54]	X *	[55]
			Cynomolgus macaque (*Macaca fascicularis*)	X	[56]		
			Rhesus macaque (*Macaca mulatta*)	X	[54]		
			Mandrill (*Mandrillus sphinx*)			X *	[20]
			Hamadryas baboon (*Papio hamadryas*)	X	[51]		
		Hominidae	Western lowland gorilla (*Gorilla Gorilla Gorilla*)			X *	[36,37]
Rodentia	Myomorpha	Cricetidae	Bank vole (*Clethrionomys glareolus*)	X	[57]		
			Chinese hamster (*Cricetulus griseus*)	X	[58]		
			Syrian hamster (*Mesocricetus auratus*)	X	[59]	X *	[60]
			Deer mouse (*Peromyscus maniculatus*)	X	[61]		
		Muridae	House mouse (*Mus musculus*)	X	[62]		
Scandentia		Tupaiidae	Northern tree shrew (*Tupaia belangeri*)	X	[63]		
Sirenia		Trichechidae	West Indian manatee (*Trichechus manatus*)			X *	[64]

Background colors indicate species’ orders. X indicates that an infection in this category has been reported. The asterisk (*) indicates that the SARS-CoV-2 infection occurred in captivity. The ‘^=^’ indicates that the status of captivity is unknown.

**Table 2:** An overview of SARS-CoV-2 clinical signs across species in cases in zoological institutions.

Species	Hippopotamus (*Hippopotamus amphibius*)	Canadian Lynx (*Lynx canadensis*)	Lion (*Panthera leo*)	Tiger (*Panthera tigris*)	Snow Leopard (*Panthera uncia*)	Leopard Cat (*Prionailurus bengalensis*)	Fishing Cat (*Prionailurus viverrinus*)	Cougar (*Puma concolor*)	White-Nosed Coati (*Nasua narica*)	Binturong (*Arctictis binturong*)	Western Lowland Gorilla (*Gorilla Gorilla*)
**Number of Animals** (*n*)	3	4	37	24	6	1	1	2	2	2	12
**Sex** (Male/Female/NA)	0/3/0	0/0/5	13/12/12	6/11/7	1/2/3	1/0/0	1/0/0	0/1/1	1/1/0	1/1/0	1/4/7
**Age in Years** Average (Range)	25.0 (14–41)	NA	9.1 (2–20)	8.8 (4–15)	5.3 (1–9)	1.0 (1)	5.0 (5)	12 (12)	4 (4)	9 (8–10)	31.4 (35–47)
**Symptoms** % (Yes/No/NA)	100.0% (3/0/0)	100.0% (4/0/0)	96.4% (27/1/9)	95.0% (19/1/4)	83.3% (5/1/0)	100.0% (1/0/0)	100.0% (1/0/0)	100.0% (2/0/0)	0.0% (0/2/0)	50.0% (1/1/0)	91.7% (11/1/0)
**Duration of Clinical Signs in Days** Average (Range)	17 (17)	25.8 (17–35)	14.8 (1–49)	10.2 (1–32)	NA	2 (2)	NA	22.5 (22–23)	NA	NA	10.4 (1–14)
**Coughing** % (Yes/No/NA or Asymptomatic)	0.0% (0/3/0)	75.0% (3/1/0)	81.5% (22/5/10)	94.7% (18/1/5)	60.0% (3/2/1)	0.0% (0/1/0)	0.0% (0/1/0)	50.0% (1/1/0)	NA (0/0/2)	0.0% (0/1/1)	90.9% (10/1/1)
**Sneezing** % (Yes/No/NA or Asymptomatic)	0.0% (0/3/0)	0.0% (0/4/0)	25.9% (7/20/10)	5.3% (1/18/5)	0.0% (0/5/0)	100.0% (1/0/0)	0.0% (0/1/0)	0.0% (0/2/0)	NA (0/0/2)	0.0% (0/1/1)	0.0% (0/11/1)
**Wheezing** % (Yes/No/NA or Asymptomatic)	0.0% (0/3/0)	0.0% (0/4/0)	14.8% (4/23/10)	63.2% (12/7/5)	40.0% (2/3/1)	0.0% (0/1/0)	0.0% (0/1/0)	0.0% (0/2/0)	NA (0/0/2)	0.0% (0/1/1)	0.0% (0/11/1)
**Ocular Discharge** % (Yes/No/NA or Asymptomatic)	0.0% (0/3/0)	0.0% (0/4/0)	11.1% (3/24/10)	15.8% (3/16/4)	0.0% (0/5/1)	0.0% (0/1/0)	0.0% (0/1/0)	0.0% (0/2/0)	NA (0/0/2)	0.0% (0/1/1)	0.0% (0/11/1)
**Nasal Discharge** % (Yes/No/NA or Asymptomatic)	66.7% (2/1/0)	0.0% (0/4/0)	37.0% (10/17/10)	36.8% (7/12/5)	0.0% (0/5/1)	100.0% (1/0/0)	0.0% (0/1/0)	100.0% (2/0/0)	NA (0/0/2)	0.0% (0/1/1)	100.0% (11/0/1)
**Lethargy** % (Yes/No/NA or Asymptomatic)	33.3% (1/2/0)	100.0% (4/0/0)	55.6% (15/12/10)	63.2% (12/7/5)	20.0% (1/4/1)	0.0% (0/1/0)	100.0% (1/0/0)	0.0% (0/2/0)	NA (0/0/2)	100.0% (1/0/1)	41.7% (5/7/1)
**Hyporexia** % (Yes/No/NA or Asymptomatic)	33.3% (1/2/0)	100.0% (4/0/0)	37.0% (10/17/10)	52.3% (10/9/5)	40.0% (2/3/1)	0.0% (0/1/0)	100.0% (1/0/0)	100.0% (2/0/0)	NA (0/0/2)	100.0% (1/0/1)	72.7% (8/3/1)
**Vomiting** % (Yes/No/NA or Asymptomatic)	0.0% (0/3/0)	0.0% (0/4/0)	0.0% (0/27/10)	5.3% (1/18/5)	0.0% (0/5/1)	0.0% (0/1/0)	0.0% (0/1/0)	0.0% (0/2/0)	NA (0/0/2)	0.0% (0/1/1)	0.0% (0/11/1)
**Diarrhea** % (Yes/No/NA or Asymptomatic)	0.0% (0/3/0)	25% (1/3/0)	0.0% (0/27/10)	5.3% (1/18/5)	20.0% (1/4/1)	0.0% (0/1/0)	100.0% (1/0/0)	100.0% (2/0/0)	NA (0/0/2)	0.0% (0/1/1)	0.0% (0/11/1)
**Epistaxis** % (Yes/No/NA or Asymptomatic)	0.0% (0/3/0)	0.0% (0/4/0)	7.1% (2/26/9)	10.5% (2/17/5)	0.0% (0/5/1)	100.0% (1/0/0)	0.0% (0/1/0)	0.0% (0/2/0)	NA (0/0/2)	0.0% (0/1/1)	0.0% (0/11/1)
**Death** % (Yes/No/NA or Asymptomatic)	33.3% (1/2/0)	0.0% (0/4/0)	5.6% (2/34/0)	0.0% (0/24/0)	0.0% (0/5/1)	0.0% (0/1/0)	0.0% (0/1/0)	0.0% (0/2/0)	0.0% (0/2/0)	0.0% (0/1/1)	0.0% (0/11/1)
**Reference**	[13,14,65]	[31]	[2,28,31,32,33,34,35,36,37,38,39]	[2,28,31,34,37,41,42,43,44,66]	[28,45]	[37]	[28]	[33]	[28]	[28]	[36,37]

Background colors indicate the data category: gray = general information on reported cases, blue = information on clinical signs, green = references. NA is used to indicate that the data is not available for this species.

**Table 3:** An overview of transmission characteristics reported across species in cases in zoological institutions.

Species	Hippopotamus (*Hippopotamus amphibius*)	Canadian Lynx (*Lynx canadensis*)	Lion (*Panthera leo*)	Tiger (*Panthera tigris*)	Snow Leopard (*Panthera uncia*)	Leopard Cat (*Prionailurus bengalensis*)	Fishing Cat (*Prionailurus viverrinus*)	Cougar (*Puma concolor*)	White-Nosed Coati (*Nasua narica*)	Binturong (*Arctictis binturong*)	Western Lowland Gorilla (*Gorilla Gorilla*)
**Number of Animals** (*n*)	3	4	37	24	6	1	1	2	2	2	12
**Sex** (Male/Female/NA)	0/3/0	0/0/5	13/12/12	6/11/7	1/2/3	1/0/0	1/0/0	0/1/1	1/1/0	1/1/0	1/4/7
**Age in Years** Average (Range)	25.0 (14–41)	NA	9.1 (2–20)	8.8 (4–15)	5.3 (1–9)	1.0 (1)	5.0 (5)	12 (12)	4 (4)	9 (8–10)	31.4 (35–47)
**Intermittent shedding** % (Yes/No/NA)	100.0% (2/0/1)	NA (0/0/4)	77.8% (7/2/28)	75.0% (6/2/16)	100.0% (6/0/0)	NA (0/0/1)	100.0% (1/0/0)	NA (0/0/2)	0.0% (0/2/0)	0.0% (0/2/0)	NA (0/0/12)
**Animal Vaccinated** % (Yes/No)	0.0% (0/3)	0.0% (0/4)	8.1% (3/34)	8.3% (2/22)	50.0% (3/3)	0.0% (0/1)	100.0% (1/0)	0.0% (0/2)	100.0% (2/0)	100.0% (2/0)	0.0% (0/12)
**Keeper Vaccinated** % (Yes/No/NA)	100.0% (2/0/1)	NA (0/0/5)	100.0% (5/0/32)	NA (0/0/24)	NA (0/0/6)	NA (0/0/1)	NA (0/0/1)	NA (0/0/2)	NA/ (0/0/2)	NA/ (0/0/2)	100.0% (7/0/5)
**Keepers use PPE** % (Yes/No/NA)	100.0% (1/0/2)	NA (0/0/5)	60.9% (14/9/14)	50.0% (9/9/6)	NA (0/0/6)	100.0% (1/0/0)	NA (0/0/1)	NA (0/0/2)	NA/ (0/0/2)	NA/ (0/0/2)	100.0% (12/0/0)
**Reference**	[13,14,65]	[31]	[2,28,31,32,33,34,35,36,37,38,39]	[2,28,31,34,37,41,42,43,44,66]	[28,45]	[37]	[28]	[33]	[28]	[28]	[36,37]

Background colors indicate the data category: gray = general information on reported cases, blue = information on transmission characteristics, green = references. NA is used to indicate that the data is not available for this species.

**Table 4:** An overview of reported SARS-CoV-2 diagnostic methods across species in cases in zoological institutions.

Species	Hippopotamus (*Hippopotamus amphibius*)	Canadian Lynx (*Lynx canadensis*)	Lion (*Panthera leo*)	Tiger (*Panthera tigris*)	Snow Leopard (*Panthera uncia*)	Leopard Cat (*Prionailurus bengalensis*)	Fishing cat (*Prionailurus viverrinus*)	Cougar (*Puma concolor*)	White-Nosed Coati (*Nasua narica*)	Binturong (*Arctictis binturong*)	Western Lowland Gorilla (*Gorilla Gorilla*)
**Number of Animals** (*n*)	3	4	37	24	6	1	1	2	2	2	12
**Sex** (Male/Female/NA)	0/3/0	0/0/5	13/12/12	6/11/7	1/2/3	1/0/0	1/0/0	0/1/1	1/1/0	1/1/0	1/4/7
**Age in Years** Average (Range)	25.0 (14–41)	NA	9.1 (2–20)	8.8 (4–15)	5.3 (1–9)	1.0 (1)	5.0 (5)	12 (12)	4 (4)	9 (8–10)	31.4 (35–47)
**Serology** % (Positive/Negative/NA)	100.0% (1/0/1)	NA (0/0/4)	100.0% (12/0/25)	100.0% (10/0/14)	NA (0/0/6)	NA (0/0/1)	NA (0/0/1)	NA (0/0/2)	NA (0/0/2)	NA (0/0/2)	0.0% (0/1/11)
**Days Between Onset of Clinical Signs and Serology** (Range)	70 (70)	NA	72.0 (1–195)	109.5 (6–196)	NA	NA	NA	NA	NA	NA	NA
**Rapid Antigen Detection Test (RADT)** % (Positive/Negative/NA)	NA (0/0/3)	NA (0/0/4)	100.0% (5/0/32)	NA (0/0/24)	NA (0/0/6)	NA (0/0/1)	NA (0/0/1)	NA (0/0/2)	NA (0/0/2)	NA (0/0/2)	NA (0/0/12)
**Fecal PCR** % (Positive/Negative/NA)	100.0% (2/0/1)	25.0% (1/3/0)	72.0% (18/7/12)	100.0% (16/0/8)	100.0% (3/0/3)	100.0% (1/0/0)	NA (0/0/1)	NA (0/0/2)	NA (0/0/2)	NA (0/0/2)	100.0% (5/0/7)
**Fecal PCR Ct-value** Average (Range)	37.8 (35.0–39.2)	28.8 (28.8)	29.7 (11.6–40.0)	30.5 (14.3–40.0)	30.4 (28.1–34.0)	26.1 (26.1)	NA	NA	NA	NA	32.4 (29.7–33.1)
**Pooled Fecal Sample** (Yes/No/NA)	0/2/1	0/4/0	2/24/11	66.7% (16/0/8)	0/3/3	0/1/0	NA (0/0/1)	NA (0/0/2)	NA (0/0/2)	NA (0/0/2)	0/5/7
**Days Between Positive Fecal PCR Tests** Average (Range)	16.5 (12–21)	NA	20.0 (3–40)	11.7 (2–24)	10.3 (2–22)	NA	NA	NA	NA	NA	8 (8)
**Rectal Swab PCR** % (Positive/Negative/NA)	NA	NA	100.0% (10/0/27)	100.0% (1/0/23)	NA (0/0/6)	NA (0/0/1)	NA (0/0/1)	NA (0/0/2)	NA (0/0/2)	NA (0/0/2)	NA (0/0/12)
**Rectal Swab PCR Ct-value** Average (Range)	NA	NA	28.5 (16.2–34.5)	29.3 (28.2–30.4)	NA	NA	NA	NA	NA	NA	NA
**Nasal PCR** % (Positive/Negative/NA)	100.0% (2/0/1)	NA (0/0/4)	100.0% (19/0/18)	85.7% (6/1/17)	NA (0/0/6)	NA (0/0/1)	NA (0/0/1)	100.0% (1/0/1)	NA (0/0/2)	NA (0/0/2)	100.0% (2/0/10)
**Nasal PCR Ct-value** Average (Range)	30.0 (15.6–40.0)	NA	25.1 (16.9–39.5)	23.3 (17.7–29.0)	NA	NA	NA	NA	NA	NA	24.2 (19.7–33.7)
**Days Between Positive Nasal PCR Tests** (Range)	23.5 (13–34)	NA	13.8 (3–33)	NA	NA	NA	NA	29 (29)	NA	NA	NA
**Oral PCR** % (Positive/Negative/NA)	NA (0/0/3)	NA (0/0/4)	100.0% (1/0/36)	50.0% (2/2/20)	NA (0/0/6)	NA (0/0/1)	NA (0/0/1)	NA (0/0/2)	NA (0/0/2)	NA (0/0/2)	NA (0/0/12)
**Oral PCR Ct-value** (Range)	NA	NA	37.2 (37.2)	27.7 (27.7)	NA	NA	NA	NA	NA	NA	NA
**Days Between Positive Oral PCR Tests** (Range)	NA	NA	20.0 (20)	NA	NA	NA	NA	NA	NA	NA	NA
**Necropsy** (Yes/No/NA)	1/0/2	NA (0/0/4)	1/0/36	NA (0/0/24)	NA (0/0/6)	NA (0/0/1)	NA (0/0/1)	NA (0/0/2)	NA (0/0/2)	NA (0/0/2)	NA (0/0/12)
**Tissue PCR Ct-value** Average (Range)	31.8 (26.7-37.0)	NA	NA	NA	NA	NA	NA	NA	NA	NA	NA
**Virus Isolation** % (Positive/Negative/NA)	NA (0/0/3)	NA (0/0/4)	55.6% (5/4/28)	75.0% (6/2/16)	NA (0/0/6)	NA (0/0/1)	NA (0/0/1)	NA (0/0/2)	NA (0/0/2)	NA (0/0/2)	NA (0/0/12)
**SARS-CoV-2 Variant** (Non-variant/Alpha/Delta/NA)	0/0/2/1	0/0/4/0	3/12/28/0	8/6/10/0	3/0/3/0	0/1/0/0	0/0/1/0	2/0/0/0	0/0/2/0	0/0/2/0	0/5/7/0
**Intermittent Shedding** (Yes/No/NA)	100.0% (2/0/1)	NA (0/0/4)	77.8% (7/2/28)	75.0% (6/2/16)	100.0% (6/0/0)	NA (0/0/1)	100.0% (1/0/0)	NA (0/0/2)	0.0% (0/2/0)	0.0% (0/2/0)	NA (0/0/12)
**Animal Vaccinated** % (Yes/No)	0.0% (0/3)	0.0% (0/4)	8.1% (3/34)	8.3% (2/22)	50.0% (3/3)	0.0% (0/1)	100.0% (1/0)	0.0% (0/2)	100.0% (2/0)	100.0% (2/0)	0.0% (0/12)
**Keeper Vaccinated** % (Yes/No/NA)	100.0% (2/0/1)	NA (0/0/5)	100.0% (5/0/32)	NA (0/0/24)	NA (0/0/6)	NA (0/0/1)	NA (0/0/1)	NA (0/0/2)	NA/ (0/0/2)	NA/ (0/0/2)	100.0% (7/0/5)
**Keepers use PPE** % (Yes/No/NA)	100.0% (1/0/2)	NA (0/0/5)	60.9% (14/9/14)	50.0% (9/9/6)	NA (0/0/6)	100.0% (1/0/0)	NA (0/0/1)	NA (0/0/2)	NA/ (0/0/2)	NA/ (0/0/2)	100.0% (12/0/0)
**Reference**	[13,14,65]	[31]	[2,28,31,32,33,34,35,36,37,38,39]	[2,28,31,34,37,41,42,43,44,66]	[28,45]	[37]	[28]	[33]	[28]	[28]	[36,37]

Background colors indicate the data category: gray = general information on reported cases, blue = information on transmission characteristics, green = references. NA is used to indicate that the data is not available for this species.

## Data Availability

Not applicable.
